# Pemphigoid Gestationis in a Third Trimester Pregnancy

**DOI:** 10.1155/2014/127628

**Published:** 2014-11-09

**Authors:** Şenol Şentürk, Nursel Dilek, Yeşim Bayoğlu Tekin, Sabri Çolak, Betül Gündoğdu, Emine Seda Güvendağ Güven

**Affiliations:** ^1^Department of Obstetrics and Gynecology, Faculty of Medicine, Recep Tayyip Erdoğan University, Merkez, Islampasa Mahallesi, Sehitler Caddesi, No. 74, 53100 Rize, Turkey; ^2^Department of Dermatology, Faculty of Medicine, Recep Tayyip Erdoğan University, Merkez, 53100 Rize, Turkey; ^3^Department of Pathology, Faculty of Medicine, Atatürk University, Merkez, 25000 Erzurum, Turkey

## Abstract

Pemphigoid gestationis (PG) is a rare vesiculobullous dermatosis of pregnancy. It is commonly seen in second or third trimester. The diagnosis is frequently made with direct immunofluorescence studies of perilesional skin. Prompt recognition and appropriate management may reduce morbidity of this disease. Herein we present a case of pemphigoid gestationis occurring in a 33-year-old primigravida woman with symptoms of generalized pruritus.

## 1. Introduction

Pemphigoid gestationis (PG) is a rare, self-limited autoimmune bullous skin disorder of pregnancy, with an incidence varying from 1 : 50.000 to 60.000 pregnancies depending on the prevalence of the HLA-haplotypes DR3 and DR4 [1, 2]. The term “herpes gestationis” was first used by Milton in 1872. When healthcare professionals (general practitioners and other specialists) are not aware of this condition, it is likely that the pregnant woman will not be treated appropriately and this will probably lead to a preterm birth and neonatal pemphigoid gestationis. The aim of this communication is to increase awareness among clinicians of the diagnosis of PG in a pregnant woman presenting with itching.

## 2. Case Report

**Figure 1:**
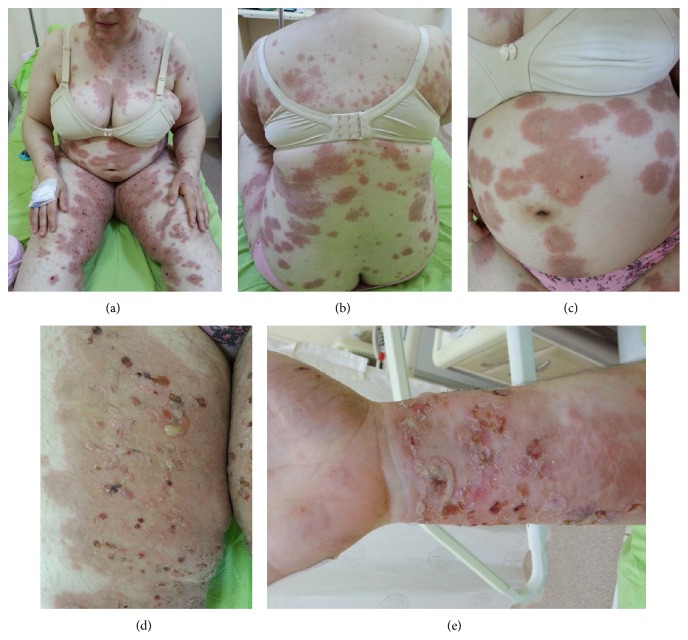
Macroscopic skin lesions of a 33-week pregnant woman with pemphigoid gestationis. (a) Macular, confluent, pruritic lesion in the abdomen and arms. (b) On the back. (c) On the abdomen. (d) Many tense and collapsed bullae on the leg. (e) Collapsed bullae on the forearm.

**Figure 2:**
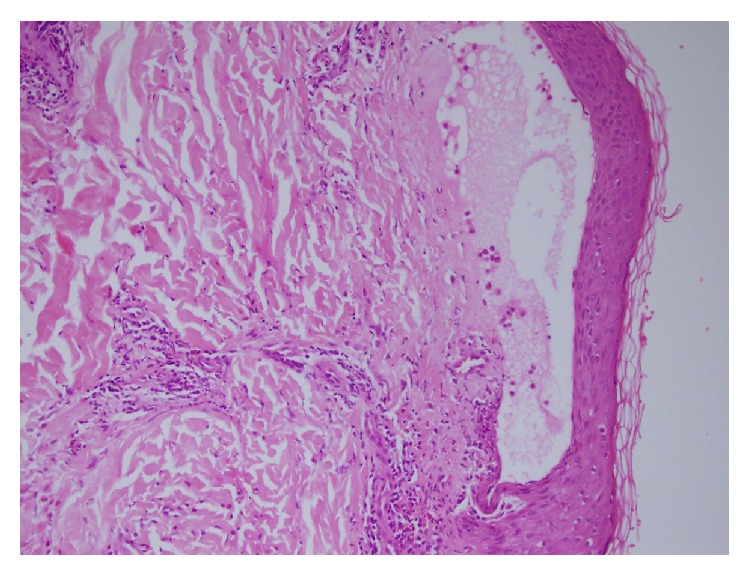
Cutaneous biopsy in lesional skin. The hematoxylin and eosin staining shows dermal-epidermal detachment, with cellular infiltration extending to the dermis (H and E, ×200).

**Figure 3:**
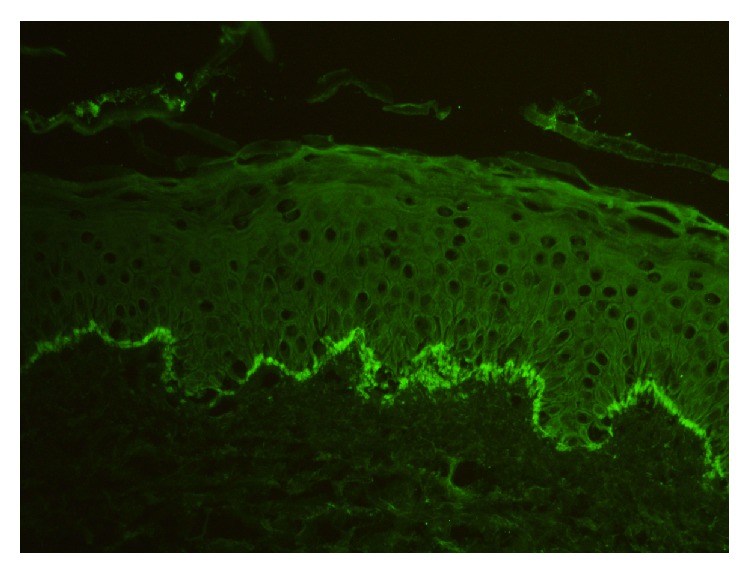
Direct immunofluorescence photomicrograph showing linear band of complement C3 deposition along the basement membrane zone (×200).

**Figure 4:**
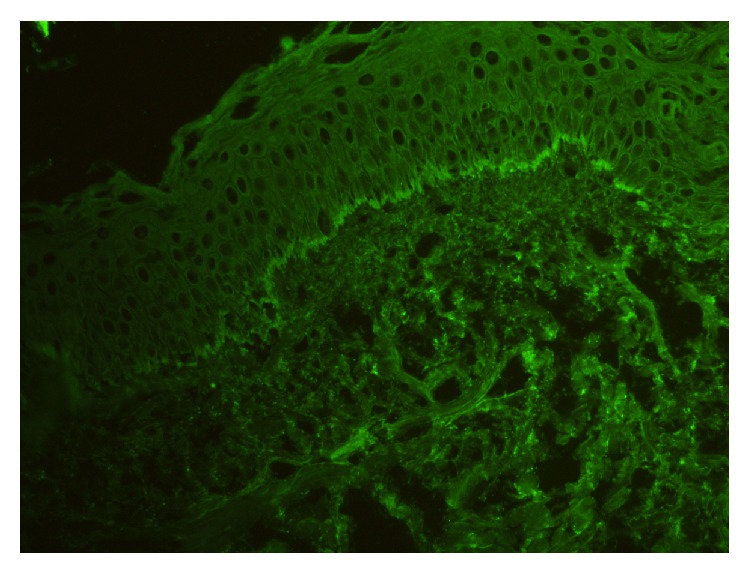
Direct immunofluorescence photomicrograph showing linear band of poor IgG deposition along the basement membrane zone (×200).

A 33-year-old primigravida woman in her third trimester of pregnancy presented to the obstetrics department with severe itching. Examination of the patient confirmed a single, live intrauterine pregnancy of 33 gestational weeks. A widespread erythematous rash was also evident, with tense fluid-filled blisters accompanied by severe itching. The patient indicated these symptoms had begun two weeks previously. The patient was referred to the dermatology polyclinic, where examination showed erythematous maculae scattered over the arms, legs, trunk, and neck (Figures 1(a), 1(b), 1(c), 1(d), and 1(e)), some with burst and crusted tense blisters on them. The patient's personal history was unremarkable. Routine hemogram and biochemical tests produced normal results, and vitamin B12, folic acid, thyroid hormones, and antibody levels were all within normal ranges. Anti-BP 180-NC16 could not be obtained because this test could not be carried out in the biochemical laboratory of our hospital. Subepidermal disintegration was detected by means of punch biopsy (Figure 2). Direct immunofluorescence showed linear C3 deposition (Figure 3) and weak IgG deposition (Figure 4) at the basement-membrane zone. A diagnosis of PG was made from these findings. Systemic corticosteroid treatment was initiated at a dose of 40 mg/day prednisolone. Since a regression was observed in the lesions of the patient after two weeks, the prednisolone dose was reduced gradually. No new blister formation was observed during hospitalization. Prednisolone treatment was started at a dose of 10 mg/day on the 15th day of hospitalization and the patient was then discharged. The patient had a mild relapse after 10 days, prednisolone dose was increased to 20 mg/day, and an improvement was observed. The dose was reduced after one week and maintained at 10 mg/day. The remaining follow-up period of the pregnancy was uneventful and the patient gave birth to a live baby measuring 3600 g in weight and 50 cm in length through vaginal delivery in the 39th gestational week. No abnormality was detected at the newborn examination. Treatment was maintained until 6 weeks after delivery.

## 3. Discussion

PG has a similar pathogenesis with bullous pemphigoid, involving autoantibodies directed against the NC16A (noncollagenous) domain of bullous pemphigoid antigen 2. The underlying pathophysiology of PG remains to be clarified. Nevertheless, it is hypothesized that major histocompatibility complex (MHC) class II antigens, present within the placenta, may induce an immune response that cross-reacts with maternal skin [3, 4]. An increased incidence of PG has also been reported in individuals that carry human leukocyte antigen- (HLA-) DR3 and HLA-DR4 [3].

Typically PG occurs in the second or third trimester of pregnancy, or rarely during the immediate postpartum period, and characteristically regresses within weeks to months (3 months) after delivery [5]. PG usually begins with intense itchy erythematous urticarial papules and plaques surrounding the umbilicus. The lesions quickly spread to the abdomen, back, chest and extremities, mucosal surfaces, palms, and soles of the feet, but sparing the face. Subsequently, clear liquid filled blisters are usually formed, but these may be absent in some of the cases [4]. Also in our case, the lesions occurred in the third trimester, similar to cases in the literature.

PG differs from other perinatal pruritic dermatoses such as pruritic urticarial papules and plaques of pregnancy. Diagnosis of PG is dependent on three key symptoms as follows.

(a) Tense subepidermal blisters; (b) the presence of complement C3 and/or immunoglobulin G deposition along the basement membrane zone under direct immunofluorescence; and (c) serum anti-BP180-NC16 antigen [6]. In one study it was indicated that, in patients with PG, autoantibodies against BP 180 were identified in 93% of cases by immunoblotting and in 86.3% by ELISA [7]. Histopathologic analysis revealed the presence of subepidermal bulla including numerous eosinophils. The presence under DIF (direct immunofluorescence) of a linear deposition of complement component protein C3, with or without IgG, along the basement membrane zone of perilesional skin, is an essential component for the diagnosis of PG. Depositions of C3 and IgG are also seen in the placenta and fetal skin [8]. Although indirect immunofluorescence reveals the presence of circulating IgG autoantibodies in 20–60% of cases, a complement fixation test demonstrates the presence of these specific autoantibodies in 90% of PG patients [5, 9]. DIF is gold standard for the diagnosis of PG [2, 10]. Also in our case, on DIF investigation, a linear deposition of C3 and a deposition of IgG were detected along the basement membrane zone of perilesional skin.

Treatment depends on the stage and severity of the disease and aims to prevent blister formation and control pruritus. Topical corticosteroids, with and without oral antihistamines, may be sufficient to treat the mild cases [1, 11]. More serious cases cannot be treated with topical corticosteroids alone; therefore potent topical glucocorticoids, oral corticosteroids (prednisone 0.5~1 mg/kg/day), and oral antihistamines are reserved for such cases [12]. Approximately 10% of all neonates born to affected mothers with PG may have skin lesions that resemble PG [13]. The disease in newborns is thought to result from the mother's autoantibodies which are passively transferred to the fetus, but it could also be caused by resting maternal hormones [5]. Typically, the skin lesions of PG regress spontaneously in the first months [14]. In our case, histological examination and direct immunofluorescence, carried out because of clinical suspicion of PG, confirmed the diagnosis. Systemic treatment was initiated with methylprednisolone at a dose of 0.5 mg/kg/day during the pregnancy. A gradual clinical improvement was observed, despite a relapse after the first attempt to reduce the dosage. This favourable condition resulted in the extension of the systemic treatment until delivery and for a further 6 weeks thereafter. An asymptomatic healthy male infant was born without skin lesions.

In conclusion, PG is a rare autoimmune disease that causes blistering of the skin. It is a clinical entity which should always be taken into consideration in the differential diagnosis of itchy vesiculobullous skin diseases seen in pregnancy. In the management of pregnant women with PG, gynecologists, dermatologists, pathologists, and pediatricians should collaborate in a multidisciplinary approach to the prevention or treatment of maternal, fetal, and neonatal complications.
